# Interaction of the Spo20 Membrane-Sensor Motif with Phosphatidic Acid and Other Anionic Lipids, and Influence of the Membrane Environment

**DOI:** 10.1371/journal.pone.0113484

**Published:** 2014-11-26

**Authors:** Habib Horchani, Maud de Saint-Jean, Hélène Barelli, Bruno Antonny

**Affiliations:** Institut de Pharmacologie Moléculaire et Cellulaire, Université de Nice Sophia Antipolis et CNRS, Valbonne, France; Simon Fraser University, Canada

## Abstract

The yeast protein Spo20 contains a regulatory amphipathic motif that has been suggested to recognize phosphatidic acid, a lipid involved in signal transduction, lipid metabolism and membrane fusion. We have investigated the interaction of the Spo20 amphipathic motif with lipid membranes using a bioprobe strategy that consists in appending this motif to the end of a long coiled-coil, which can be coupled to a GFP reporter for visualization in cells. The resulting construct is amenable to *in vitro* and *in vivo* experiments and allows unbiased comparison between amphipathic helices of different chemistry. *In vitro*, the Spo20 bioprobe responded to small variations in the amount of phosphatidic acid. However, this response was not specific. The membrane binding of the probe depended on the presence of phosphatidylethanolamine and also integrated the contribution of other anionic lipids, including phosphatidylserine and phosphatidyl-inositol-(4,5)bisphosphate. Inverting the sequence of the Spo20 motif neither affected the ability of the probe to interact with anionic liposomes nor did it modify its cellular localization, making a stereo-specific mode of phosphatidic acid recognition unlikely. Nevertheless, the lipid binding properties and the cellular localization of the Spo20 alpha-helix differed markedly from that of another amphipathic motif, Amphipathic Lipid Packing Sensor (ALPS), suggesting that even in the absence of stereo specific interactions, amphipathic helices can act as subcellular membrane targeting determinants in a cellular context.

## Introduction

The identity of cellular membranes results from the combination of specific determinants such as rare phosphoinositides and small G proteins [1], [2], and bulk physicochemical properties [3], [4]. Among them are electrostatics, which depends on abundant anionic lipid such as phosphatidylserine (PS) [5], [6], and lipid packing, which is related to lipid geometry and membrane curvature [4], [7]. Thanks to the use of lipid mass spectroscopy [8], [9], of high throughput analysis of membrane proteins [10] and of specific lipid-binding protein domains [6], [11], important advances have been made in our understanding of the features that distinguish cellular membranes and which are used by peripheral proteins for their proper targeting [4].

One lipid that remains mysterious in a cellular context is phosphatidic acid (PA) [12], [13]. PA harbors the simplest polar headgroup among all phospholipids and it is unclear how a mere esterified phosphate can act as a specific ligand for peripheral proteins. Furthermore, depending on its neighboring membrane environment, PA bears either one or two negative charges [13]. In cells PA is involved in at least two different processes. PA is a key intermediate in phospholipid synthesis (the Kennedy pathway) and also a second messenger: the product of phosphatidylcholine (PC) hydrolysis by phospholipases D (PLD1 and PLD2). Because the Kennedy pathway occurs at the endoplasmic reticulum whereas PLDs are recruited to other organelles and notably the plasma membrane, different pools of PA probably coexist in the cell [12]. Tracking these pools is difficult and there is clearly a need for better readouts of PA in a cellular context [14]; hence the interest for protein regions that have been reported to sense PA [15], [16], [17]. Such regions have been identified in various proteins such as lipin 1 and the transcriptional repressor Opi1 whose localization between cellular membranes and the nucleus is controlled by PA levels [18], [19], [20]. As expected, these regions are rich in basic residues. However, there is no consensus domain or sequence for the general recognition of PA by proteins.

**Figure 1:**
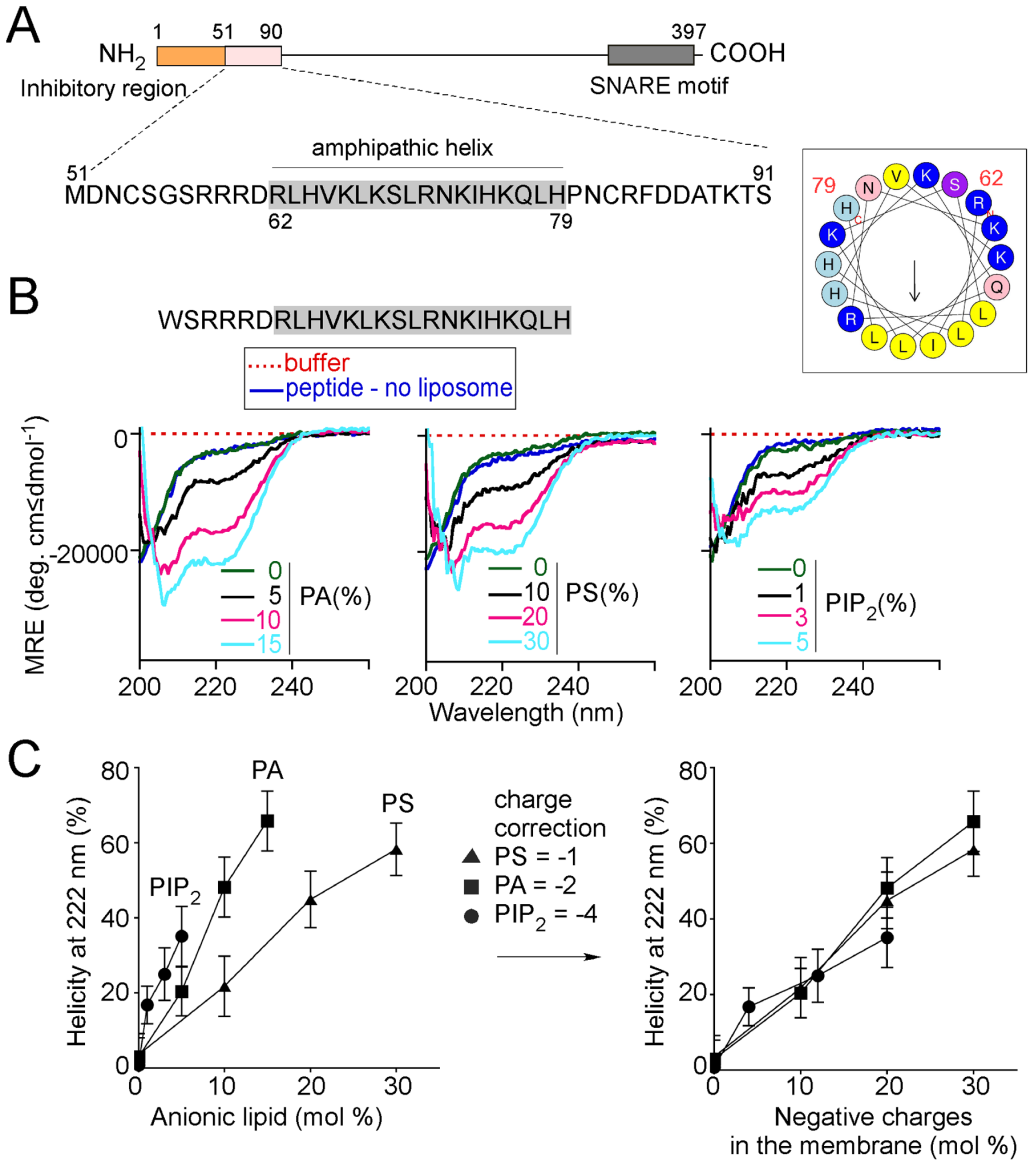
Folding of the membrane sensor region of Spo20 onto anionic liposomes. (**A**) Domain organization of Spo20. The sequence of the membrane-binding region is indicated. The helical wheel representation, which was drawn using heliquest (http://heliquest.ipmc.cnrs.fr/) [28], illustrates the amphipathic character of its central part. Hydrophobic residues are shown in yellow, arginines and lysines in dark blue, histidines in light blue, glutamine and asparagine in pink and serine in purple. (**B**) Far-UV CD spectra of the Spo20 peptide (aa 56–79; 30 µM) in solution (dark blue curve) or with 3 mM of small liposomes (Rh = 15±4 nm) containing increasing % of either PA, PS, or PIP_2_ (black to cyan curves). The remaining lipids in the liposome were (in mol %) PE (25), and PC (the concentration of which varied from 75 to 45% depending on the concentration of anionic lipid). MRE, mean residue ellipticity. (**C**) Left: helicity at 222 nm as a function of the percentage of PA, PS or PIP_2_ in the liposomes and as determined from the spectra shown in panel B. Right: helicity at 222 nm as a function of the amount of negative charges carried by PA, PS or PIP_2_ in the liposomes.

Spo20 is a member of the SNARE (Soluble NSF Attachment Protein Receptor protein) family and is necessary to mediate spore formation in *Saccharomyces cerevisiae*. The formation of spores requires an unusual cell division event in which daughter cells are formed within the cytoplasm of the mother one. Spo20 controls membrane fusion events allowing yeast cells to construct a prospore membrane around meiotic nuclei [21]. Spo20 differs from most SNAREs by lacking a transmembrane segment (Figure 1A). Instead, two N-terminal regions upstream of the fusogenic region regulate its cellular localization [22]. The most N-terminal region (1–50) controls the translocation of Spo20 from the nucleus to the cytosol at the onset of meiosis, whereas the adjacent region (51–91) directly targets Spo20 to the prospore. The function of the long segment between this region and the SNARE domain is not known. GFP constructs derived from the 51–91 sequence localize preferentially to the prospore membrane [23]. Intriguingly their localization is affected by genetic manipulations of the PA level but not of two other highly charged anionic lipids, phosphatidylinositol-4-phosphate (PIns(4)P) and phosphatidylinositol-4,5-bisphosphate (PIP_2_) [23]. Therefore this region (51–91) has been proposed to act as a sensor of PA. In agreement with this hypothesis, *in vitro* experiments show that Spo20 binds to liposomes enriched in PA [23].

In this study, we used various approaches to further address the lipid binding properties of the Spo20 membrane sensor region. Our results indicate that the 51–91 region of Spo20 undergoes a disordered to alpha-helix transition at the surface of PA-enriched lipid membranes. However, membrane binding and helix folding also occurred in the presence of other anionic lipids. Moreover, binding of the Spo20 membrane sensor region to anionic liposomes was unaffected by extensive mutations that change drastically the sequence of the helix while keeping its physical chemistry constant. Importantly, these mutations also did not affect the subcellular localization of a bioprobe derived from the Spo20 membrane sensor region. We conclude that the targeting of the Spo20 membrane sensor region to subcellular lipid membranes is mostly driven by non-stereospecific electrostatic interactions.

## Materials and Methods

### Protein construction, expression and purification

The Spo20 fragment (aa 51–91) (gift from Nicolas Vitale) fused to GMAP210 aa 39–375 [24], [25], [26], [27] was cloned via *BamH*I restriction site into a pGEX-4T-2 expression vector (Pharmacia), which includes a GST tag and a thrombin cleavage site at the N-terminus. The endogenous Cys present in the coiled coil region were mutated by site-directed mutagenesis QuikChange kit (Stratagene) according to [27]. In order to obtain more soluble and thrombin-cleavable fractions of this Spo20-GMAP coiled coil (GCC) fusion, the coiled coil of GMAP was shortened by including a stop codon at position K202. All plasmids constructed in this study were confirmed by sequencing.

The Spo20-GCC (39–201) and the mutants (invSpo20-GCC and swapSpo20-GCC) constructs were expressed in *E. coli* at 23°C in the presence of 1 mM IPTG (at O.D. 600 nm = 0.8) overnight. All purification steps were conducted in Buffer A (50 mM Tris HCl, pH 7.4, 120 mM NaCl) supplemented with a cocktail of protease inhibitors (1 mM PMSF, 1 mM pepstatin, 10 mM bestatin, 10 mM phosphoramidon) and 1 mM DTT. Cells were lysed with a French press and the lysate was ultra-centrifuged at 30.000 rpm for 30 min. The supernatant was incubated for 2 h with glutathione-Sepharose 4B beads (Amersham). After 3 washing steps with freshly degassed buffer A containing no DTT, the beads were incubated with thrombin to cleave the GST fusion and allow the release of the protein of interest.

Spo20-Artificial coiled coil (ACC1)-mCherry was constructed in a pmCherryN1 vector from a synthetic gene encoding both the Spo20 51–91 sequence and an artificial coiled sequence. InvSpo20-ACC2 with a GFP at the C terminus was constructed in a pEGFP-N1 vector from a synthetic gene encoding both the inverted Spo20 sequence as well as a second artificial coiled-coil sequence, ACC2, that could not heterodimerize with ACC1. For details about the construction of bioprobes based on artificial coiled-coil (ACC1 and ACC2) and their exact nucleotide and amino-acid sequence see Appendix S1.

### NBD labeling of Spo20

NBD labeling of Spo20-GCC and its mutant swapSpo20-GCC, both displaying two endogenous Cys residues (Cys54 and Cys82) flanking the spo20 sensor region, was performed as described for the N-terminal region of GMAP210 [24], [25], [26], [27] using a 10-fold molar excess of N,N′-dimethyl-N-(iodoacetyl)-N′-(7-nitrobenz-2-oxa-1,3-diazol-4-yl)ethylenediamine (IANBD amide, Molecular Probes) in dimethylformamide (DMF volume <5%). After 5 minutes at room temperature the reaction was stopped by the addition of 10 mM Cystein and the sample was applied to a NAP-5 column to separate protein from excess probe. Aliquots before and after the reaction were analyzed by SDS/PAGE. The gel was directly visualized in a fluorescence imaging system (FUJI LAS-3000) to detect NBD-labeled of Spo20-CC or swapSpo20-CC and then stained with Sypro Orange to determine the purity of proteins. The percentage of labeling was then estimated from the optical density (OD) at 280 and 495 nm.

### Liposomes

Lipids in chloroform were purchased from Avanti Polar Lipids except egg phosphatidylcholine (PC; Sigma). Unless otherwise stated, the liposomes contained (in mol %): liver phosphatidylethanolamine (PE; 25), cholesterol (25), various amounts of brain phosphatidylserine (PS; 0 to 30), 1-palmitoyl-2-oleoyl-sn-glycero-3-phosphate (PA; 0 to 15) and/or liver phosphatidyl-inositol-(4,5)bisphosphate (PIP_2_; 0 to 10%). The remaining lipid was PC. Liposomes used in flotation experiments contained 0.2 mol % NBD-PE (3(N-(7-nitrobenz-2-oxa-1,3-diazol-4-yl)-PE; Molecular Probes). Liposomes used to follow the recruitment of NBD-labeled proteins contained no fluorescent probe.

A dried film was prepared by evaporation of lipids in chloroform in a rotary evaporator. The film was resuspended in 50 mM Hepes, pH 7.2, 120 mM K-acetate (HK buffer) giving a suspension of large multilamellar liposomes. The suspension was then frozen and thawed 5 times (using liquid nitrogen and a water bath) and then extruded through 0.2 µm polycarbonate filters using a mini-extruder (Avanti Polar Lipids, Inc.). For experiments with liposomes of varying curvature, the liposomes were extruded sequentially through polycarbonate filters of decreasing pore size (0.2, 0.1, 0.05 and 0.03 µm).

For CD spectroscopy, very small liposomes were prepared by sonication with a Microson sonicator (XL2000, New York). Lipid debris were removed by centrifugation at 45.000 rpm for 20 min to produce a homogenous population of liposomes. The liposome radius was estimated by dynamic light scattering (DLS) using a Dyna Pro instrument. Liposomes were stored at room temperature and used within 2 days after preparation.

### Flotation experiments

Proteins (0.75 µM) and liposomes (0.75 mM containing 0.2% NBD-PE) were first mixed in 50 mM Hepes, pH 7.2, 120 mM K-acetate and 1 mM MgCl_2_ (HKM buffer) at room temperature for 5 min in a total volume of 150 µl. Thereafter the sample was adjusted to 30% sucrose by mixing 100 µl of a 60% (w/v) sucrose solution in HK buffer supplemented with 1 mM MgCl_2_ (HKM buffer) and covered with two cushions of decreasing density (200 µl of 25% sucrose in HKM and 50 µl of HKM). After 1 h centrifugation at 45 000 rpm and 20°C in a Beckman swing rotor (TLS-55), the bottom, middle, and top fractions were collected using a Hamilton syringe and analyzed by SDS-PAGE using Sypro Orange (Molecular Probes) staining and a FUJI LAS-3000 fluorescence imaging system.

### NBD fluorescence

NBD-labeled Spo20-GCC and the mutants (0.3 µM) were incubated at 30°C with liposomes (0.35 mM) of defined radius in HKM buffer supplemented with 1 mM DTT. Emission fluorescence spectra were measured from 520 to 680 nm (bandwidth of 5 nm) upon excitation at 505 nm (bandwidth of 3 nm) in a small quartz cell (total volume 250 µl) in a Shimadzu RF 5301-PC fluorimeter. Control spectra were acquired in the absence of protein to subtract the light scattering signal from the liposomes. For kinetics measurements, NBD fluorescence was continuously measured at 530 nm (bandwidth 5 nm) upon excitation at 505 nm (bandwidth 3 nm). The sample initially contained 350 µM liposomes in HKM buffer (total volume 600 µl) and was placed in a cylindrical quartz cell, which was continuously stirred with a small magnetic bar and thermostated at 30°C. At the indicated times, NBD-labeled Spo20-GCC and PLD were injected from stock solutions through a guide in the cover of the fluorimeter adapted to Hamilton syringes, such as to not interrupt the fluorescence recording.

### Circular dichroism measurements

CD measurements were performed on a Jasco J-815 spectropolarimeter. The Spo20 synthetic peptide: WSRRRDRLHVKLKSLRNKIHKQLH was from Sigma. The N-terminal W residue was introduced to facilitate concentration determination by UV absorption. The experiments were performed at room temperature in buffer containing 150 mM KCl and 10 mM Tris HCl, pH 7.5 using a thin quartz cell with an optical path length of 0.05 cm. Each spectrum is the average of several scans recorded from 195 to 260 nm, with a bandwidth of 1 nm, a step size of 0.5 nm and a scan speed of 50 nm.min^−1^. The buffer contribution was subtracted. The alpha-helix content was calculated from the molar ellipticity at 222 nm ([∂]_222 nm_) according to: % alpha-helix = −([∂]_222 nm_+2340)/303.

### Cell transfection

Telomerase-immortalized human retinal pigment epithelial (RPE1) cells were grown in DMEM/F12 glutaMAX medium (Invitrogen) supplemented with 10% calf foetal serum and penicillin and streptomycin. Cells were transfected with 4 µg DNA/well with Lipofectamine2000 (Life Technologies) according to manufacturer's protocol for for 24 h and fixed in 3% paraformaldehyde during 20 min in PBS, permeabilized with 0.5% saponin for 10 min in PBS followed by treatment for 1 hour with 10% horse serum and 0.05% saponin in PBS to avoid non-specific labeling. The cells were examined under a Leica SP5 confocal microscope.

## Results

### CD spectroscopy of the Spo20 membrane sensor region

The region of Spo20 that is necessary and sufficient for its localization at the prospore membrane lies between amino acids 51 and 91 (Figure 1A) [23]. Within this region, a stretch of 18 amino acids (62–79) plays the most critical role [23]. Because a helical projection of this sequence shows a clear segregation between hydrophobic and polar residues [28], it has been suggested that it could form an amphipathic alpha-helix (Figure 1A) [23]. However, the structure of the Spo20 sensor region has never been addressed experimentally.

We performed circular dicroïsm (CD) experiments with a peptide corresponding to the Spo20 57–79 aa sequence and containing an additional N-terminal tryptophan residue to facilitate titration by UV spectroscopy (Figure 1B). In solution the CD spectrum of the peptide indicated no marked secondary structure. In the presence of phosphatidylcholine (PC) liposomes the spectrum remained unchanged suggesting no significant folding on neutral membranes. In contrast, with increasing amounts of PA in the liposomes, the spectrum of the Spo20 peptide showed the characteristic shape of an a-helix with two minima at 208 and 222 nm. With 15 mol% PA present in the liposomes, we estimated that ≈65±7% of the peptide adopted an a-helical conformation.

Next, we tested two other anionic lipids, phosphatidylserine (PS) and phosphatidyl-inositol-(4,5)bisphosphate (PIP_2_). These lipids also promoted a clear coil to α-helix transition (Figure 1B). Plotting the CD intensity at 222 nm as a function of the mol% of the anionic lipids suggested that the Spo20 sensor region binds strongly to PIP_2_, moderately to PA and weakly to PS. However, the PIP_2_>PA>PS preference might reflect the differences in charges carried by these lipids: PS and PIP_2_ have one and four negative charges, respectively, whereas PA carries one or two negative charges depending on the environment and notably phosphatidylethanolamine (PE), which favors the fully deprotonated form [13]. We corrected the dose-response curves by taking into account the net charge of the corresponding polar heads. This correction made the three dose responses very similar (Figure 1C). Therefore, the Spo20 membrane sensor region seems quite promiscuous in its binding to anionic lipids.

CD spectroscopy requires large amounts of peptide (30 µM in the experiments of Figure 1). To minimize crowding effects at the membrane surface, we used a 100∶1 mol∶mol lipid∶protein ratio (i.e. 3 mM lipids). At this high lipid concentration and because large liposomes strongly scatter light, the use of very small liposomes obtained by sonication is mandatory to preserve a good signal∶noise ratio. However, the extreme curvature of these liposomes (radius in the 15 nm range as estimated by dynamic light scattering) might create a bias by favoring a-helix penetration in the distorted bilayer [29], [30]. Consequently, putative differences between anionic lipids for the adsorption of the Spo20 lipid-binding region might be minimized on these liposomes. We thus looked for alternative approaches to determine whether the Spo20 lipid-binding region is specific for PA in a less permissive membrane environment and notably on less curved lipid bilayers.

### Incorporation of Spo20 amphipathic region in a bioprobe

An ideal system to study the lipid membrane binding properties of various amphipathic helices should: (i) permit comparison between different amphipathic helices; (ii) be amenable to both *in vitro* and *in vivo* experiments, and (iii) allow quantitative binding measurements on liposomes of various size and composition.

**Figure 2:**
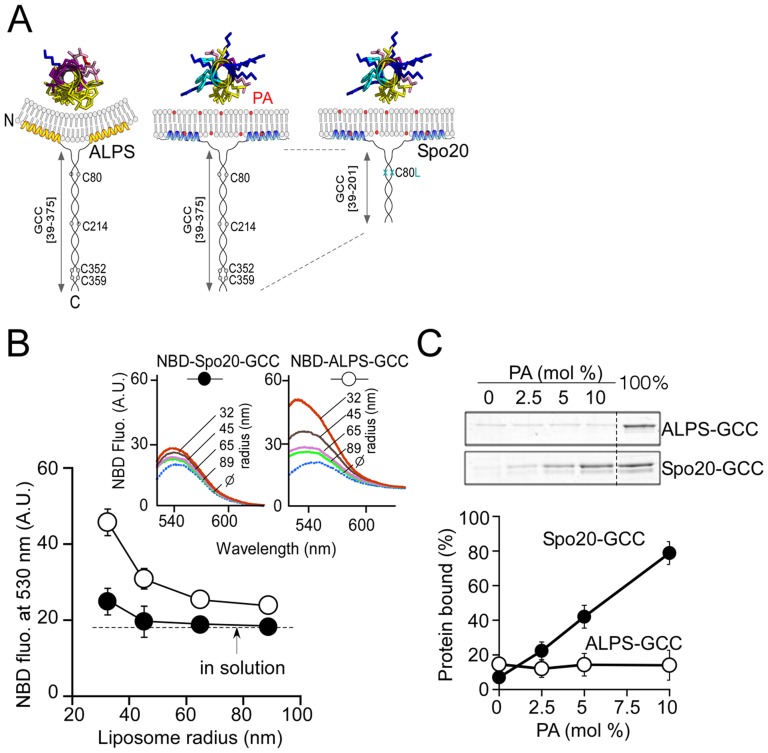
A bioprobe strategy to assess the membrane binding properties of the Spo20 sensor region. (**A**) Probe design. The probes were constructed according to the design of the N-terminal region of the Golgin GMAP-210. The ALPS motif of GMAP-210 was replaced by the Spo20 membrane region. In the case of the Spo20 probe, the coiled coil of GMAP was shortened by including a stop codon at position K202 and its endogeneous cysteines were mutagenized. The obtained construction, Spo20-GCC, is more soluble and was used for flotation and NBD-fluorescence experiments. (**B**) Membrane partitioning of the NBD labeled forms of Spo20-GCC or ALPS-GCC (0.3 µM) in the presence of liposomes of defined size as obtained by extrusion (0.35 mM lipids). The liposomes contained (mol %): PC (40), PE (30), cholesterol (25) and PA (5). (**C**) Flotation experiments. Spo20-GCC but not ALPS-GCC, bound to PA containing liposomes. Spo20-GCC or ALPS-GCC (0.75 µM) was incubated without liposomes or with liposomes (0.75 mM lipids) containing (mol %): PE (25), cholesterol (25), PS (15) and increasing amount of PA (0 to 15). The remaining lipid was PC. Liposomes were extruded through 0.2 µm filters.

We used a strategy inspired by the design of a protein named GMAP-210 (Figure 2A). This long coiled coil protein, which decorates the *cis* side of the Golgi apparatus, harbors an N-terminal Amphipathic Lipid Packing Sensor (ALPS) motif [24], [26]. ALPS motifs are amphipathic helices, but their chemistry is very different from Spo20. *In vitro*, ALPS motifs recognize the curvature of weakly charged or neutral liposomes [25], [31]. In vivo, they interact with transport vesicles of the early secretory pathway [4]. Replacing the ALPS motif of the N-terminal region of GMAP-210 by the sensor region of Spo20 should create a bioprobe with novel lipid-membrane binding properties. We previously used this approach to compare ALPS motifs and the amphipathic helical region of alpha-synuclein [27].

The design illustrated in Figure 2A offers several advantages. First, different amphipathic helices can be compared within the same molecular context, which is imposed by the simple coiled-coil geometry. Second, the binary nature of the probe allows concomitant membrane binding of two sensors, which might increase sensitivity. Third, the probe can be readily modified for *in vitro* and *in vivo* tests; for example by adding a fluorescent protein at the C-terminus of the coiled-coil region for visualization in cells. Last, the well-understood principles of parallel coiled-coil assembly allow constructing numerous homo- or hetero-dimeric probes with complementary features. These principles are detailed in the supporting file Appendix S1. In brief, we used three coiled-coil sequences: one from GMAP (GCC), and two artificial coiled coils (ACC1) and (ACC2), on which we fused a defined membrane sensor at the N-terminus and, when needed, a fluorescent protein at the C-terminus. For example, Spo20-GCC-GFP designed a probe containing (from N to C) the Spo20 (51–91) amphipathic region, the 39–201-sequence of GMAP210, and Green Fluorescent Protein (GFP).

**Figure 3:**
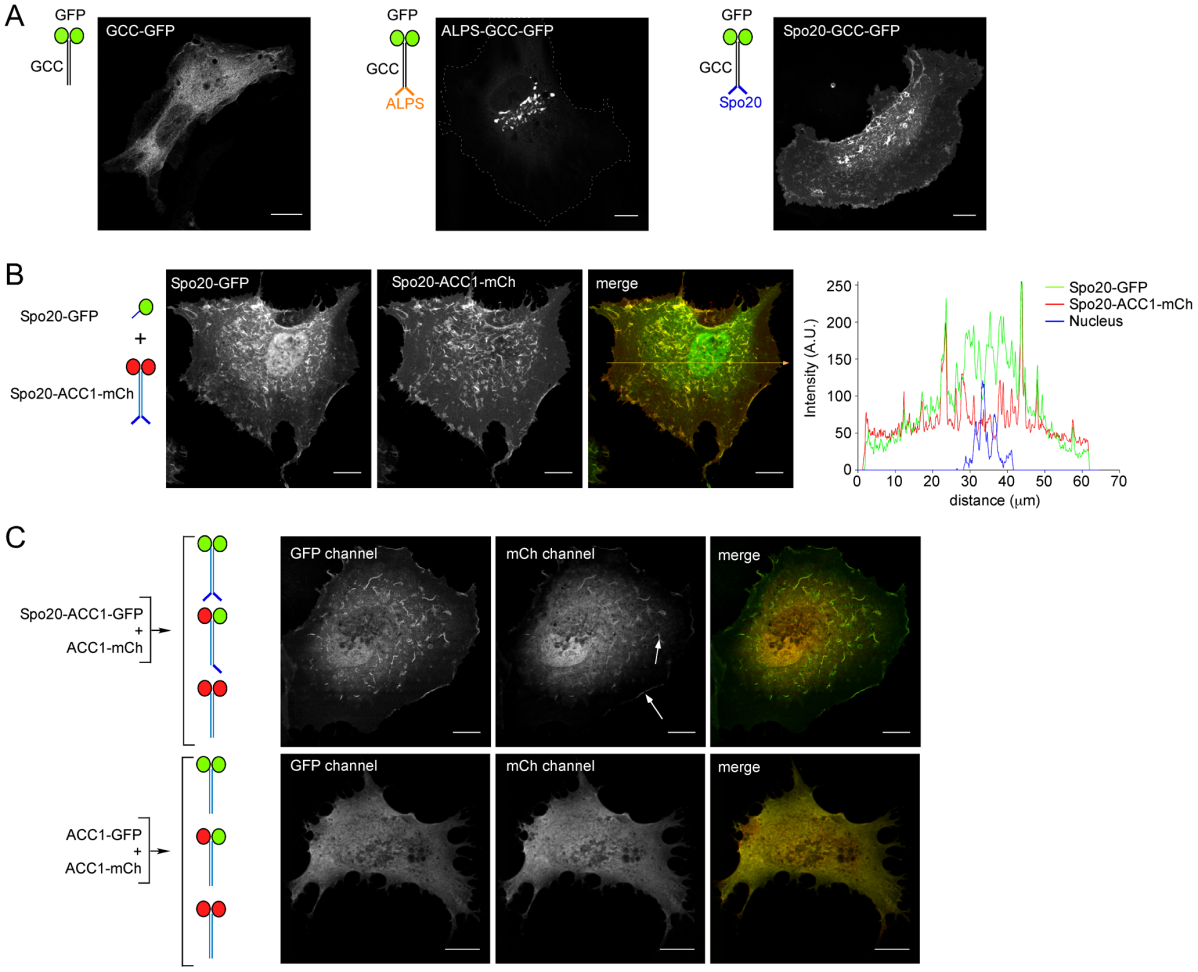
Subcellular localization of coiled-coil bioprobes containing ALPS or the Spo20 membrane sensor region. (**A**) GCC-GFP, ALPS-GCC-GFP or Spo20-GCC-GFP in RPE1 cells. (**B**) Coexpression of monomeric Spo20-GFP and Spo20-ACC1-mCherry. The two constructs labeled the same membrane structures (plasma membrane ruffles), but Spo20-GFP also invades the nucleus and is more cytosolic than Spo20-ACC1-mCherry. (**C**) Coexpression of Spo20-ACC1-GFP and ACC1-mCherry or ACC1-GFP and ACC1-mCherry. Due to dimerisation, three coiled-coil probes should form with different color properties and various numbers of Spo20 membrane sensor regions. The moderate labeling of plasma membrane ruffles by mCherry (arrows) indicates that the heterodimer Spo20-ACC1-GFP/ACC1-mCherry, which contains one Spo20 motif, binds membranes. Scale bars, 10 µm.

The membrane binding properties of the probes were assessed by two biochemical assays and one cellular assay as illustrated by the various panels of Figures 2 and 3. In the first biochemical assay (NBD fluorescence assay), we assessed the membrane partitioning of the probe by monitoring changes in the fluorescence of a covalently attached NBD group (Figure 2B). The fluorescence of NBD increases upon intercalation into a non-polar environment, such as the core of a lipid bilayer [32]. NBD labeling was performed on two endogenous Cys residues flanking the Spo20 sensor region, or, in the case of ALPS, on a Cys residue incorporated at the N-terminus of the motif [27]. To avoid NBD labeling in the coiled-coil region, the endogenous Cys present in this region were mutated by site-directed mutagenesis (Figure 2A). In the second biochemical assay (flotation assay), the probe was incubated with liposomes, which were then collected by centrifugation at the top of sucrose cushions [31], [33]. Bound proteins were analyzed by SDS-PAGE (Figure 2C). Last, we assessed the cellular localization of the probes, now appended with a fluorescent protein (GFP or mCherry), by transient expression in Retinal Pigmental Epithelium (RPE1) cells (Figure 3).

Figure 2B compared the Spo20-GCC and the ALPS-GCC probes in the NBD fluorescence assay. In agreement with a previous study [27], binding of the ALPS-GCC probe to liposomes increased with membrane curvature (i.e. when the size of the liposomes decreased). In contrast, the Spo20-GCC probe was poorly sensitive to the size of the liposomes (Figure 2B) despite the presence of PA (5 mol%).

Figure 2C shows the result of a flotation assay where Spo20-GCC or ALPS-GCC was incubated with large unilamellar liposomes obtained by extrusion through 0.2 µm filters and containing increasing mol% of PA as well as 15 mol% PS. The amount of liposome-bound Spo20-GCC increased from 10 to 80% when the mol fraction of PA in the liposomes varied from 0 to 10% (Figure 2C). In contrast, ALPS-CC remained essentially soluble.

Figure 3A compares the subcellular localization of GCC-GFP, Spo20-GCC-GFP and ALPS-GCC-GFP. The probe containing no membrane sensor region was entirely cytosolic. In agreement with previous studies, ALPS-GCC-GFP labeled the Golgi apparatus [24]. In marked contrast, the Spo20-GCC-GFP probe was localized at the plasma membrane, notably in dorsal membrane ruffles.

A mere construct in which the Spo20 sensor region was directly appended to GFP (Spo20-GFP) has been previously used in cells as a probe of PA [15]. We compared the subcellular localization of this probe to that of Spo20-ACC1-mCherry (Figure 3B). Both Spo20-GFP and Spo20-ACC1-mCherry decorated the same membrane regions. However, Spo20-GFP was also found in the nucleus and its background signal in the cytosol was higher than that of Spo20-ACC1-mCherry. Because all coiled-coils used in this study (GCC, ACC1 and ACC2) are expected to form homodimeric coiled-coils (see Appendix S1), the better partitioning of Spo20-ACC1-mCherry to membrane-bound organelles might be due to the presence of two Spo20 membrane sensor regions as compared to Spo20-GFP. To test this hypothesis, we co-expressed two coiled-coil constructs: one containing the Spo20 membrane sensor region and GFP (Spo20-ACC1-GFP), and one containing no membrane sensor region and mCherry (ACC1-mCherry). Because these constructs share the same coiled-coil region, they should form various dimeric complexes, which could be distinguished by their color properties: Spo20-ACC1-GFP/Spo20-ACC1-GFP, ACC1-mCherry/ACC1-mCherry and Spo20-ACC1-GFP/ACC1-mCherry. As shown in Figure 3C, a fraction of the mCherry signal was found on membrane regions together with GFP. In contrast, when the two constructs lacked the Spo20 membrane sensor region, both the GFP signal and mCherry signal were found in the cytosol. Thus, the hybrid probe (Spo20-ACC1-GFP/ACC1-mCherry) displays intermediate properties between the ACC1-mCherry/ACC1-mCherry homodimer, which is soluble, and the Spo20-ACC1-GFP/Spo20-ACC1-GFP homodimer, which binds strongly to membranes. Similar results were obtained with GCC instead of ACC1 bioprobes (Figure S1).

Collectively, the experiments shown in Figures 2, 3 and S1 validate the general bioprobe strategy. Swapping the Spo20 membrane sensor region for the ALPS motif of the N-terminal region of GMAP-210 makes the probe sensitive to PA *in vitro* (Figure 2C) and directs it not at the Golgi but to other membrane regions including the plasma membrane (Figure 3A). Moreover, bioprobes based on homodimeric coiled-coils and harboring two Spo20 membrane sensor regions bind better to membranes than the monomeric Spo20-GFP construct and do not invade the nucleus (Figures 3B and C).

### The Spo20 probe is very sensitive to PA but its response is not specific and depends on the bulk lipid composition

**Figure 4:**
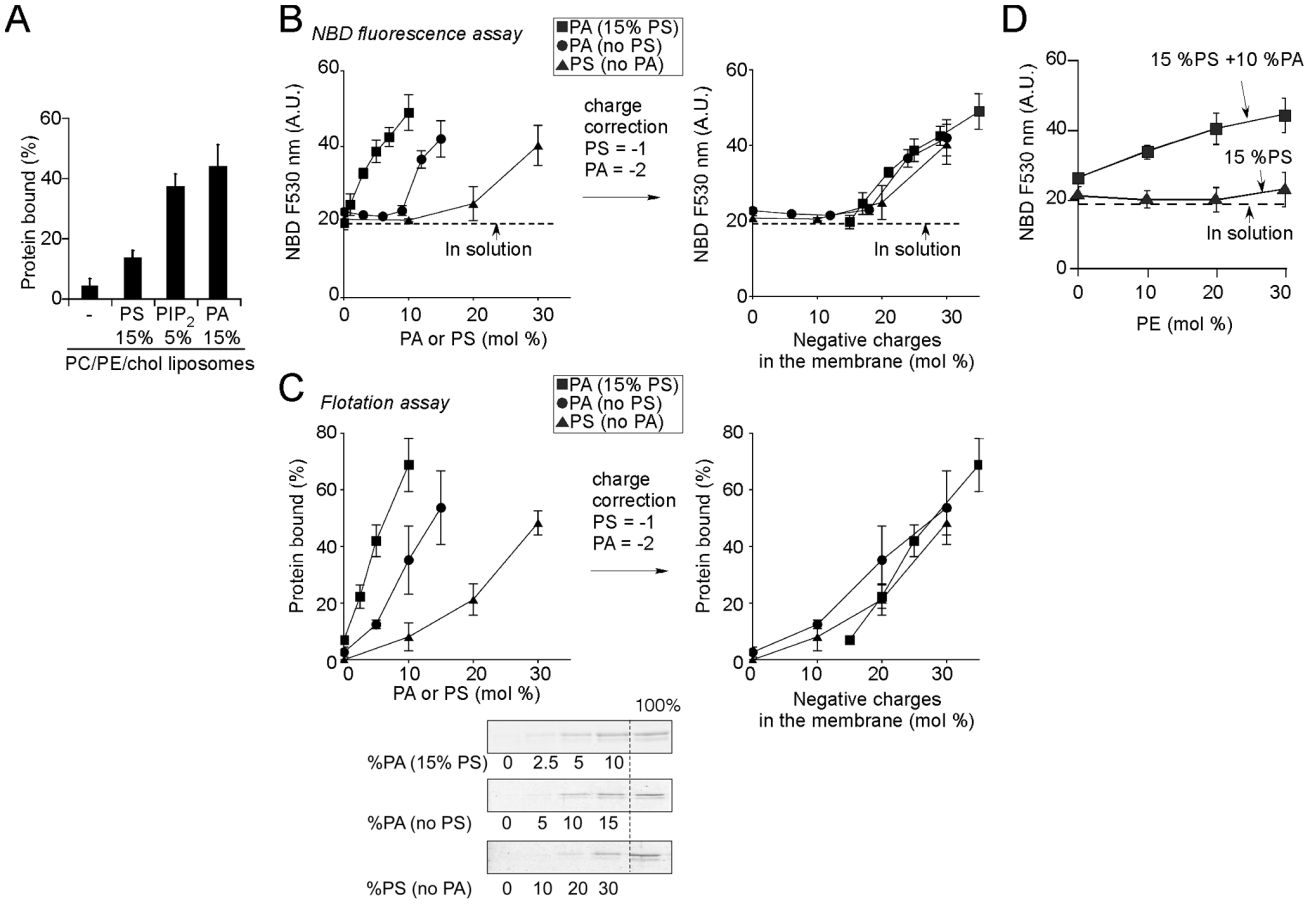
Membrane binding properties of the Spo20 bioprobe. (**A**) Spo20-GCC (0.75 µM) was incubated with or without PC/PE/Cholesterol liposomes (0.75 mM) containing (mol %) PE (25), cholesterol (25) and supplemented with PA (15), PS (30), or PIP_2_ (5). The remaining lipid was PC. Bound proteins were recovered by flotation and analyzed by SDS-polyacrylamide gel electrophoresis using Sypro orange staining. (**B, C**) Binding of Spo20-GCC to liposomes containing (mol %) PE (25), cholesterol (25) and increasing amounts of PA (circles) or PS (triangles) as indicated. The remaining lipid was PC. A dose response curve for PA with liposomes containing 25 mol % PE, 25 mol % cholesterol and 15 mol % PS is also shown (squares). Membrane partitioning was assessed either by the NBD fluorescence assay (**B**) or by the liposome flotation assay (**C**). The data shown for the NBD assay are from two independent experiments. The horizontal dashed line indicates the fluorescence level of the NBD proteins in solution. The vertical bars show the standard deviation of the NBD fluorescence intensity of Spo20-GCC. For the flotation assay, the data shown are from two or three independent experiments with different preparations of liposomes. An typical SDS gel analysis is shown. The dose response curves are shown either as a function of the mol % of PA or PS (left) or as a function of the total amount of negative charges in the membrane (right). (**D**) Effect of PE on the membrane partitioning of Spo20-GCC as assessed by NBD fluorescence. The liposomes contained (mol %) PS (15), cholesterol (25), PA (0, triangles; 10, squares) and increasing amounts of PE at the expense of PC. All liposomes were prepared by extrusion through 0.2 µm filters.

To better characterize the lipid-binding properties of the Spo20-GCC probe, we performed additional liposome binding experiments. We varied not only the nature and density of the anionic lipid present, but also the bulk lipid composition. Thus, we performed experiments with one or two anionic lipids present (e.g. PA+PS) and changed the ratio between phosphatidylcholine (PC) and phosphatidylethanolamine (PE). These experiments were motivated by the fact that the lipid environment of PA might be very different in the cell [13]; a PA molecule in the inner leaflet of the plasma membrane should be surrounded by much more PE and PS lipids than a PA molecule at the ER, where PC predominates. The results obtained from this analysis are presented in Figure 4.

In a background of PE (25 mol %), cholesterol (25 mol %) and PC, the Spo20-GCC probe bound to liposomes containing any of the 3 anionic lipids tested (PS, PIP_2_, PA). There was a preference for PA over PS. However, given the fact that PA bears two negative charges in a PE-rich environment [13], the difference between PA and PS was quite modest. Moreover, membrane binding in the presence of 5 mol% PIP_2_ was within the same range as that observed with liposomes containing 15 mol% PA (Figure 4A), which could also be due to the higher number of charges of PIP_2_ (−4) compared to PA (−2).

Next, we performed experiments with liposomes containing increasing amounts of PS or PA. The dose-response curves of the Spo20-GCC probe for PS or PA displayed a non-linear shape, an effect that was more pronounced in the NBD-fluorescence assay (Figure 4B) than in the flotation assay (Figure 4C). In the NBD-fluorescence assay, significant binding of the Spo20-GCC probe occurred above a threshold of 10 mol % PA or 20 mol% PS (Figure 4B). Adding 15 mol% PS in the liposomes shifted the PA dose-response to lower values and made the response of the probe almost linear. To determine if the apparent synergy between PS and PA reflected a preference of the probe for this combination of anionic lipids, we re-plotted the three dose-response curves (PA, PS and PA+PS) as a function of the amount of negative charges present in the liposomes. Remarkably, the corrected curves superimposed very well. We concluded that the Spo20 membrane sensor region interacts non-specifically with negatively charged lipids and that the effect of PS and PA are essentially cumulative.

In all previous experiments, PE was present in liposomes at 25 mol%. At this high level, PE promotes the deprotonation of the PA polar head, shifting its charge from −1 to −2 [13]. To test if this effect affects the binding of the Spo20 membrane sensor region, we performed NBD fluorescence experiments in the presence of liposomes containing fixed amounts of PA (0 or 10 mol%) and PS (15 mol%), and we varied the relative amounts of PC and PE (Figure 4D). In the absence of PE, the signal of the [NBD]Spo20-GCC probe was close to that observed in solution despite the presence of both PA and PS. When PE gradually replaced PC in the liposomes, the NBD signal increased up to three fold. Therefore, PE-driven deprotonation of PA strongly facilitates membrane interaction of the Spo20-GCC probe.

Taken together, the experiments shown in Figure 4 indicate that the Spo20-GCC probe does not interact specifically and stoechiometrically with PA. Instead, Spo20-GCC binds to membranes above a threshold of about 10–20 mol% of negative charges and essentially integrates the contribution of all negatively charged lipids (PA, PA and PIP_2_) present in the membrane. Furthermore, PE, which controls the ionic state of PA, improves dramatically the response of Spo20-GCC to PA.

### Real time measurements

**Figure 5:**
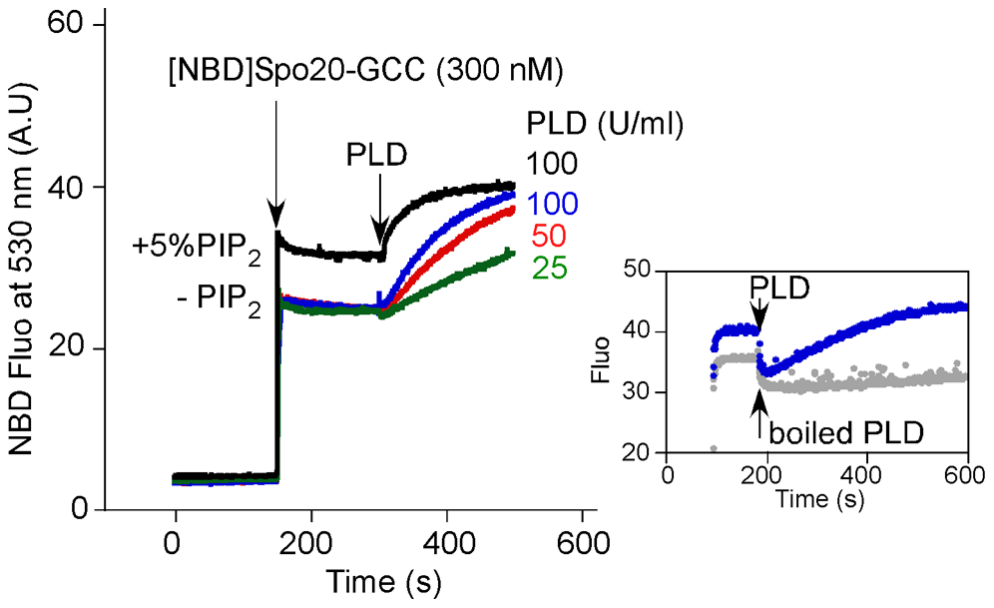
Real-time measurement of PA production by an extract containing PLD activity. The fluorescence cuvette contained liposomes (0.35 mM lipids; extrusion 0.2 µm) with 25 mol % PE, 25 mol % cholesterol supplemented or not with 5 mol % PIP_2_. The remaining lipid was PC. Then, [NBD]Spo20-GCC (0.3 µM) and a PLD extract from *Streptomyces chromofuscus* (25 to 100 U/ml) were sequentially added. Experiments were carried out in triplicates; representative traces are shown. Inset: comparison between PLD and boiled PLD under the same conditions.

In order to determine whether Spo20-GCC could detect small temporal variations in PA produced by an enzymatic activity, we performed experiments with a commercial extract containing a bacterial PLD. Initially, the sample contained liposomes of defined composition. At the indicated times, the NBD-labeled Spo20-GCC probe and the PLD extract were sequentially added (Figure 5). With liposomes containing PC (50 mol%), PE (25 mol%) and cholesterol (25 mol%), the addition of PLD caused a significant increase in the NBD signal, the rate of which was PLD concentration-dependent. Denaturating the PLD extract by boiling abolished its effect (Figure 5, inset). Interestingly, we noticed a slight delay in the signal induced by PLD, suggesting that the probe did not detect the first PA molecules produced by the enzyme (Figure 5).

Next, we repeated the experiment using liposomes supplemented wth PIP_2_ (5 mol%) to change the threshold of the probe response. Under this condition, the initial NBD fluorescence signal was higher indicating that the probe was partially bound. Furthermore, the addition of PLD caused a rapid increase in the signal, with no delay. Therefore, and in agreement with the titration experiments shown in Figure 4, the Spo20 GCC probe can be very sensitive to PA, but its response does not solely reflect the presence of this lipid and instead integrates the contribution of other lipids.

### Inverting the sequence of the Spo20 membrane sensor does not affect its localization

**Figure 6:**
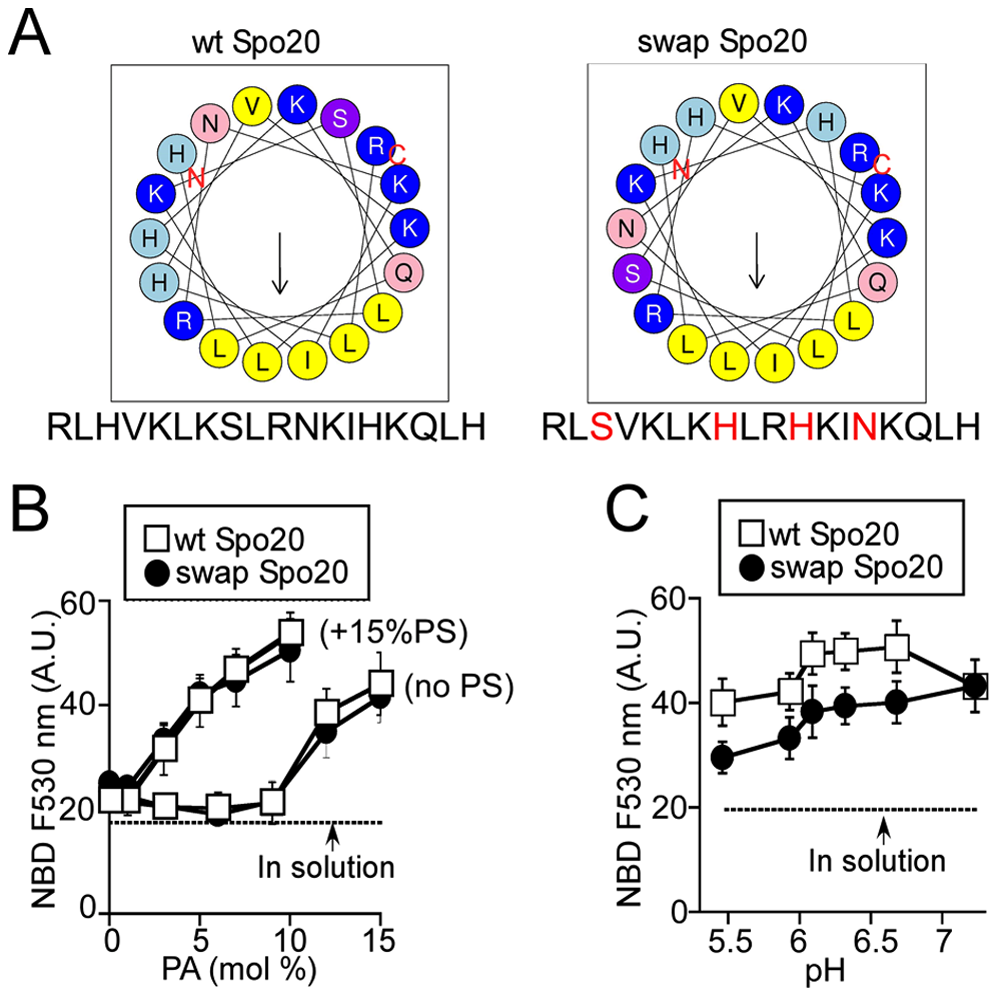
Membrane binding properties of the swap Spo20 mutant. (**A**) Helical wheel representations of the Spo20 sequence and of the corresponding swap mutant. (**B–C**) NBD fluorescence assays comparing the membrane partitioning of the [NBD]Spo20-GCC and [NBD]swapSpo20-GCC bioprobes (0.3 µM) to various liposomes (0.35 mM). In (**B**), the liposomes contained (mol %) PE (25), cholesterol (25), PS (0 or 15) and increasing amounts PA. The remaining lipid was PC. In (**C**), the liposomes contained (mol %) PC (25), PE (25), cholesterol (25), PA (10) and PS (15) and the partitioning of the constructs was tested at various pH. Data shown are mean ± S.E of 3 independent experiments. All liposomes were prepared by extrusion through 0.2 µm polycarbonate filters.

**Figure 7:**
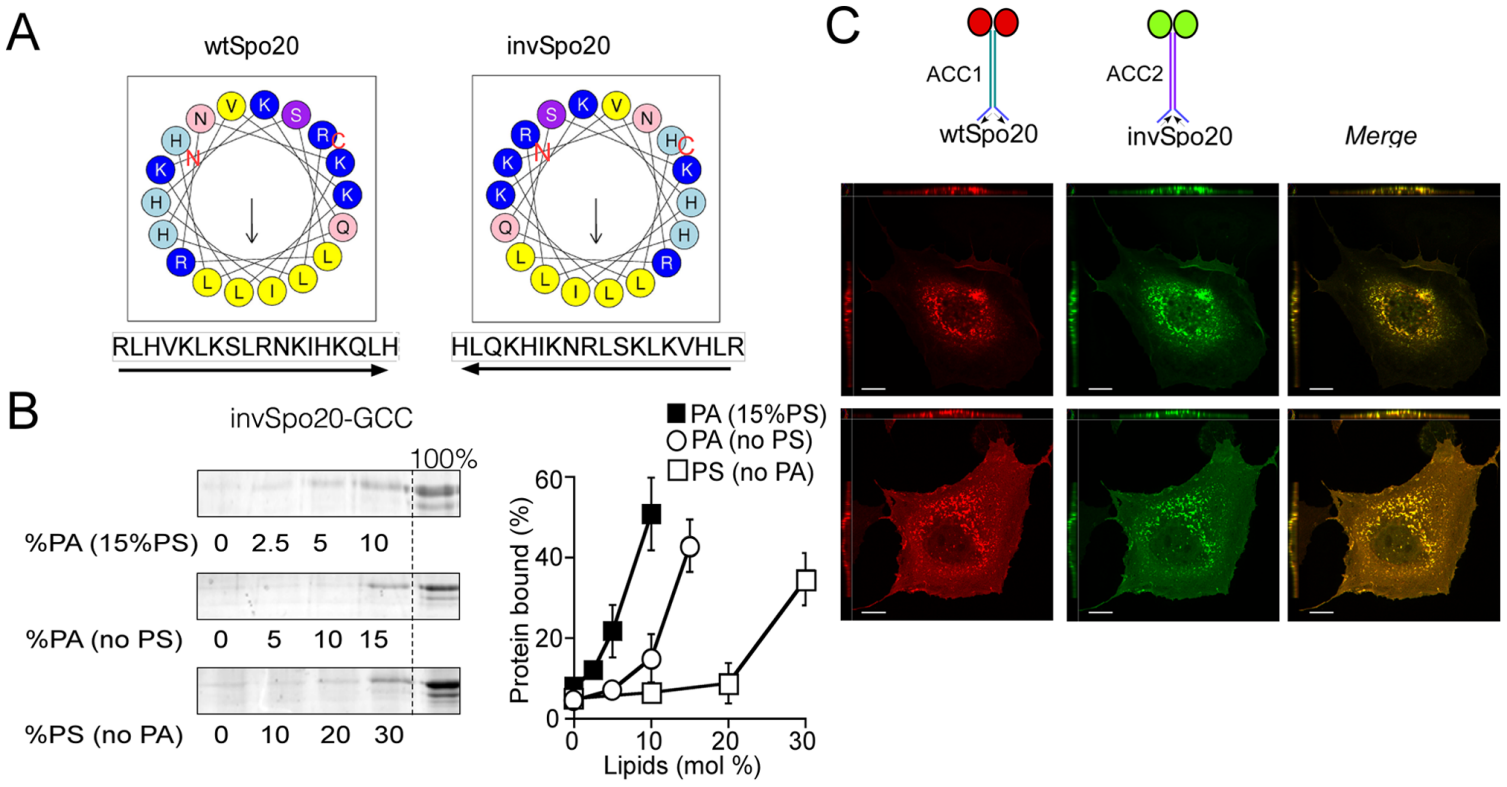
Inverting the Spo20 sequence affects neither its specificity for negatively charged lipids nor its subcellular localization. (**A**) Helical wheel representations of the Spo20 sequence and of the corresponding inverted mutant (iSpo20) where the sequence is read from the C- to the N-terminus. (**B**) Flotation Assays. Binding of iSpo20-GCC to liposomes (0.75 mM lipids; extrusion 0.2 µm) containing (mol %) PE (25), cholesterol (25), PS (0, open symbols; 15, filled symbols) and increasing amounts of PA. The experiment was repeated two or three times with different preparations of liposomes. Data show mean ± S.E of these independent experiments. (**C**) Confocal microscopy images of RPE1 cells after transfection with Spo20-ACC1-mCherry and iSpo20-ACC2-GFP. xy planes and z projections are shown. Note that the two probes co-localize almost perfectly. Scale bars = 10 µm.

A distinguishable feature of the Spo20 amphipathic helix is the presence of 3 histidines (His, Figure 1A). Histidine has a mild pKa and is usually less abundant than lysine and arginine in motifs that bind to anionic membranes [34], [35]. We reasoned that the bias towards His in the Spo20 membrane sensor region might reflect a specific requirement of this amino acid for PA recognition. To test this hypothesis, we constructed two mutants. First, we swapped two His of the amphipathic helix for two other residues of the polar face. As a result, the His residues were displaced from one side of the polar face to a more central position (swapSpo20-GCC; Figure 6A). Alternatively, we inverted the Spo20 sequence (i.e. read the sequence from the C- to the N-terminus). The sequence of this second mutant (invSpo20-GCC) differed from that of the wild-type by 8 non-conservative and 6 conservative mutations (over a total number of 18 amino acids) (Figure 7A). However and most importantly, these extensive mutations kept intact both the amino acid composition and the amphipathic character of the Spo20 membrane sensor (Figures 6A and 7A). By comparing the membrane binding properties of Spo20, swapSpo20 and invSpo20, we should sort the contribution of specific interactions with PA from non-specific interactions such as electrostatics.

We assessed the lipid binding properties of the swapSpo20-GCC construct using the NBD fluorescence assay. SwapSpo20-GCC and Spo20-GCC displayed very similar dose-responses for their binding to liposomes containing increasing amounts of PA, both in the presence and in the absence of PS (Figure 6B). The only difference between the two probes was a slight increase in the pH sensitivity of swapSpo20-GCC compared to Spo20-GCC (Figure 6C).

The invSpo20 mutant was analyzed by flotation experiments and by transient expression in RPE1 cells (Figure 7). In the flotation assay, invSpo20-GCC, displayed a clear sensitivity to PA and PS (Figure 7B). Although binding to liposomes was slightly lower than that of Spo20-GCC (compare Figures 4C and 7B), the cumulative effects of PA and PS remained very strong. Next, we cotransfected cells with two coiled-coil based probes: one bearing the wild-type Spo20 helix (Spo20-ACC1-mCherry) and one bearing the invSpo20 helix (invSpo20-ACC2-GFP). The two probes displayed different fluorescent domains as well as two different artificial coiled-coil regions (ACC1 and ACC2) to prevent the formation of heterodimers (Appendix S1). As shown in Figure 7C, Spo20-ACC1-mCherry and invSpo20-ACC2-GFP co-localized almost perfectly to cellular membranes. This observation was striking because the two probes displayed a completely different sequence. We concluded that the membrane binding properties of the Spo20 amphipathic helix is not governed by stereo-specific interactions.

### Effect of PLD2 on the localization of Spo20 bioprobes

Probes based on the Spo20 membrane sensor region are becoming widely used to follow variations in PA levels in cells [15], [16], [36], [37]. Our *in vitro* experiments suggest that the membrane sensor region of Spo20 is not a specific PA reporter because its response to anionic lipids is essentially based on electrostatic rather than specific interactions. Nevertheless, the architecture of our coiled-coil based constructs is different from that of other bioprobes. Therefore, it was important to test whether a coiled-coil based probe could respond to cellular conditions that are likely to lead to variations in the PA content of membranes.

In pilot experiments, we observed that overexpression of PLD2 did not significantly alter the distribution of homodimeric probes harboring two Spo20 membrane sensor regions. However, these probes strongly decorated the plasma membrane under normal conditions (see Figure 3). We thus hypothesized that their background binding to anionic lipids at the plasma membrane (PS, PIP_2_) might prevent detecting PA variations.

**Figure 8:**
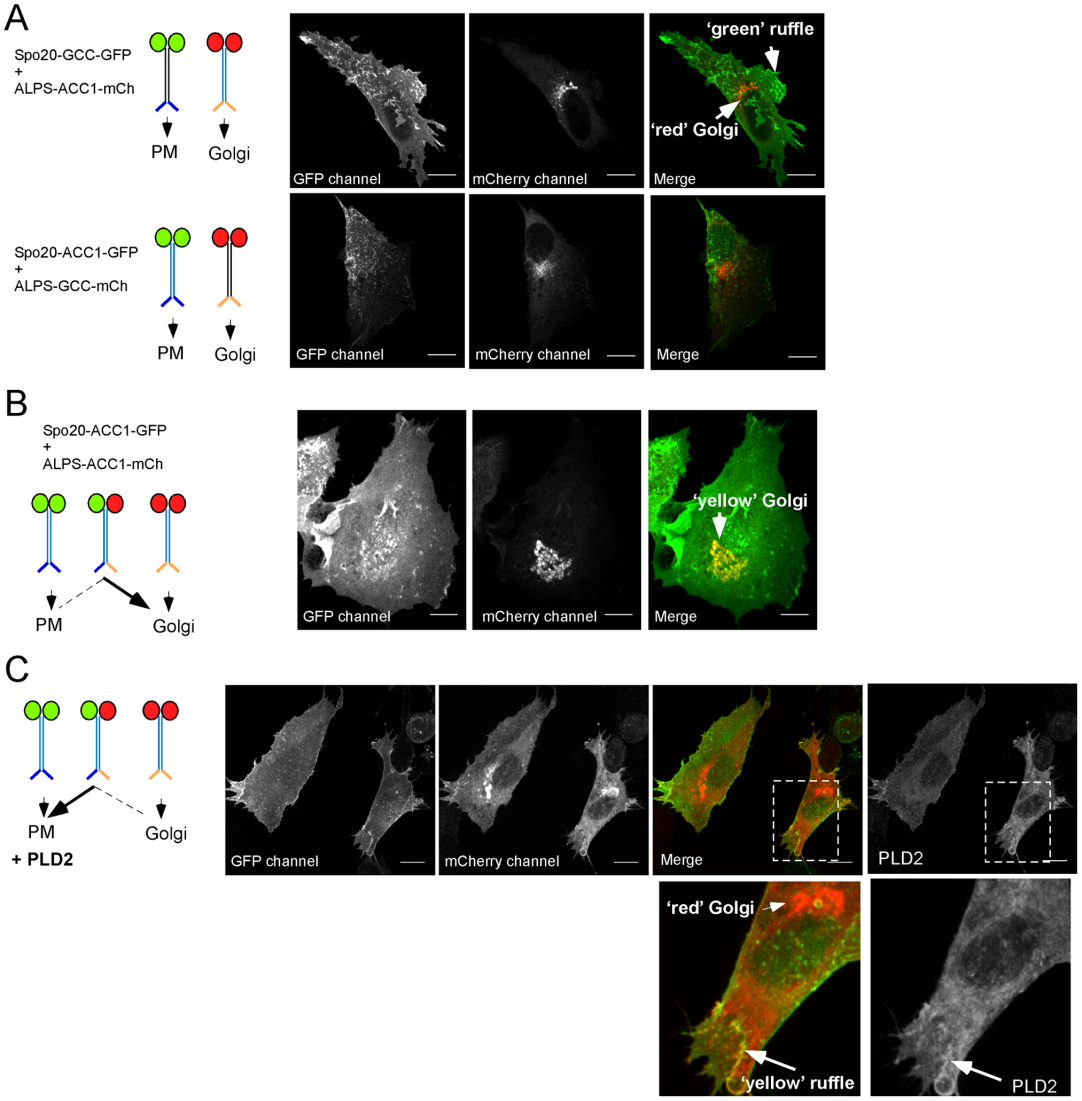
Effect of PLD2 overexpression on the subcellular localization of coiled-coil probes. (**A**) Spo20-GCC-GFP and ALPS-ACC1-mCherry were coexpressed in RPE1 cells. Because the two constructs contain different coiled-coil domains they form homodimeric probes. Spo20-GCC-GFP/Spo20-GCC-GFP stains ruffles at the plasma membranes, whereas ALPS-ACC1-mCherry/ALPS-ACC1-mCherry stains the Golgi apparatus. (**B**) Coexpression of Spo20-ACC1-GFP and ALPS-ACC1-mCherry. Because the two constructs contain the same coiled-coil, they not only homodimerize but also form the heterodimer Spo20-ACC1-GFP/ALPS-ACC1-mCherry. The dual color of the Golgi apparatus (green+red = yellow) indicates that the heterodimer is directed towards this compartment. (**C**) Same as in (**B**) but in cells overexpressing PLD2. Some plasma membrane ruffles where PLD2 localizes appear yellow, whereas the Golgi apparatus is completely red, suggesting that the Spo20-ACC1-GFP/ALPS-ACC1-mCherry heterodimer is redirected from the Golgi apparatus to the plasma membrane upon the action of PLD2. Scale bars = 10 µm.

Next, we performed experiments with heterodimeric probes carrying one ALPS motif and one Spo20 membrane sensor region. The rationale was to create a tug of war between the ALPS motif and the Spo20 membrane sensor region: the former directs coiled-coil probes towards the Golgi, whereas the latter directs coiled-coil probes towards the most charged membrane (e.g. the plasma membrane; Figures 3A and 8A). In the absence of overexpressed PLD2, coexpression of Spo20-ACC1-GFP and ALPS-ACC1-mCherry led to a ‘yellow’ Golgi apparatus, i.e. positive both for the red and in the green canals, whereas the plasma membrane was ‘green only’ (Figure 8B). This observation indicated that the ALPS motif imposed the localization of the heterodimeric Spo20-ACC1-GFP/ALPS-ACC1-mCherry probe. In contrast, cells overexpressing PLD2 displayed a red Golgi apparatus, whereas their plasma membrane displayed some yellow ruffles that corresponded to PLD2 positive regions (Figure 8C). This observation suggests that production of PA at the plasma membrane makes the Spo20 membrane sensor able to impose the subcellular localization of the heterodimeric probe at the expense of the interaction of the ALPS motif with the Golgi.

## Discussion

Apart from being defined by the segregation between polar and non-polar residues, membrane-binding amphipathic helices can be remarkably diverse in amino acid composition [38], [39], [40]. The Spo20 helix possesses a unique feature: a histidine-rich polar face. This feature, together with the fact that this motif has been proposed to recognize PA [23] prompted us to analyze its lipid-binding properties in more details. How peripheral proteins recognize PA is indeed poorly understood [12]. Furthermore, better characterizing the lipid binding properties of the Spo20 membrane sensor region is crucial considering that probes based on this sequence are becoming increasingly popular to follow the production of PA in cells [15], [36], [37].

We have found no evidence for a stereospecific interaction between PA and the Spo20 membrane sensor region. Instead, our experiments suggest that this amphipathic helix interacts non-specifically with membranes containing anionic lipids. First, the binding of the Spo20 sensor region to lipid membranes correlates with the net charge of all anionic lipid tested lipids (PA, PS and PIP_2_) (Figures 1C, 4B and 4C). Most importantly, mutations that changed drastically the sequence of the Spo20 amphipathic helix but kept its overall physical chemistry had no effect on its membrane binding properties *in vitro* (Figures 6) and preserved its subcellular localization in *vivo* (Figure 7). The most striking observation was that inverting the sequence of the Spo20 amphipathic helix had no effect despite the fact that the inverted sequence displayed only 22% identity with the original Spo20 sequence.

The lack of stereo specific interaction between the Spo20 amphipathic α-helix and PA raises a general question about the ability of amphipathic a-helices to recognize a specific lipid. Model structural studies suggest that the number of lipids in direct contact with a amphipathic helix is roughly equal to the number of amino acids that compose the helix [41]. Therefore, even a relatively short amphipathic helix such as Spo20 (18 amino-acids) can hardly recognize a single lipid while ignoring the other lipids that necessarily contact the helix. This is a fundamental difference between amphipathic a-helices and protein domains such as pleckstrin homology domains, which remain above the polar head lipid region and project a few amino acids towards a single polar head group to form a specific binding site [11]. Epsin represents an intermediate case because the specific recognition of PIP_2_ involves residues from an amphipathic helix as well as residues from the core protein domain [42].

Amphipathic helices are found in numerous cytosolic proteins that transiently interact with cellular membranes. Their availability for membrane binding is sometimes controlled by additional mechanisms (e.g. conformational change, phosphorylation, nuclear targeting), and they often act in synergy with other membrane attachment mechanisms [39]. Unbiased side-by-side comparison between amphipathic helices requires minimizing these additional mechanisms. The advantage of our bioprobe design is to place all tested amphipathic helices in a similar and favorable position for membrane interaction. Notably, the coiled-coil region should easily accommodate the crowded environment of membrane-bound organelles.

When appended to the same coiled-coil, the ALPS motif and the amphipathic helices of Spo20 target different organelles: the Golgi apparatus [24] and the plasma membrane, respectively (Figures 3A and 8A). These observations indicate a high degree of adaptation between each amphipathic helix and its host membrane, without necessarily involving specific interactions. A similar conclusion was previously obtained when comparing the localization of coiled-coil probes harboring either the alpha synuclein helix or ALPS motifs [27].

The distinguishable feature of ALPS motifs is their modest polar face composed of small and uncharged polar residues (Gly, Ser and Thr). For these helices, interaction with the lipid membrane is mostly driven by the hydrophobic effect; hence the preference of ALPS motifs for neutral membranes with lipid packing defects such as small vesicles of the early secretory pathway [4]. Compared to this extreme design, the amphipathic helix of Spo20 appears more balanced. First, both faces of the helix are well developed, making membrane curvature not a prerequisite for adsorption. Second, the preferential binding of Spo20 to the plasma membrane and to endocytic structures in transfected cells [15], [36] or to the prospore membrane in yeast [23] likely results from the strong enrichment of these membranes in anionic lipids (PS, PIP_2_ and PA), which matches the numerous cationic residues present in the Spo20 helix.

Because the Spo20 membrane sensor region can respond to small variations in PA content *in vitro* (Figure 4B and C), we do not argue that it cannot be used as a probe to detect variations in PA in a cellular context as suggested by recent careful studies [15], [36]. However, given its promiscuous binding to other anionic lipids, it is prudent to proceed with caution and include various controls to ensure that the observed effects indeed report variations in PA levels. Moreover, interaction of the Spo20 membrane sensor region with PA appears extremely sensitive to the presence of PE, which favors PA deprotonation, and to PS, which decreases the PA threshold at which the Spo20 helix binds. Therefore, Spo20-based probes seem more adapted to detect variations in PA at the inner leaflet of the plasma membrane, which is rich in PS and PE, than in other organelles and notably the ER.

## Supporting Information

Appendix S1 **Design of bioprobes with artificial coiled-coil regions.**
(PDF)

Figure S1 **Subcellular localization of GCC bioprobes containing 0, 1 or 2 Spo20 membrane sensor regions.** The experimental conditions were similar to that used in Fig. 3C except we used bioprobes containing GCC instead of ACC1 coiled-coil regions. The following pairs were coexpressed (from top to bottom): Spo20-GCC-GFP+GCC-mCherry, GCC-GFP+Spo20-GCC-mCherry, GCC-GFP+GCC-mCherry. The presence of the Spo20 membrane sensor region on one construct drives the membrane localization of the other construct. Scale bars, 10 µm.
(TIF)
