# Supplementary material for: Interaction of the Spo20 Membrane-Sensor Motif with Phosphatidic Acid and Other Anionic Lipids, and Influence of the Membrane Environment
Source: PLoS One. 2014 Nov 26;9(11):e113484. doi: 10.1371/journal.pone.0113484 (PMC4245137; doi:10.1371/journal.pone.0113484)
Supplement: Appendix S1 — Design of bioprobes with artificial coiled-coil regions. (PDF) [file pone.0113484.s001.pdf]

Appendix S1. Design of bioprobes with artificial coiled-coil regions

Supporting information file for
"Interaction of the Spo20 membrane-sensor motif with phosphatidic acid and other anionic lipids, and influence of the membrane environment" by Horchani et al.

When two coiled-coil bioprobes are co-expressed in cells, their coiled-coil regions have to be different in order to not heterodimerize. We thus designed two artificial sequences, ACC1 and ACC2, each optimized for parallel homodimerisation and not prone to heterodimerize. To this end, we followed the rules defined by Woolfson[1].

We first considered an elementary sequence of four heptads:
abcdefg abcdefg abcdefg abcdefg

By imposing *d* = L (leucine) and by introducing some asparagines (N) at position *a*, parallel dimeric coiled-coils are favored (through the formation of a leucine zipper). In addition we introduced charged residues at position *g* and *e* to promote the formation of salt bridges in the case of parallel homodimers and to induce charge repulsion in the case of heterodimers. Indeed in parallel dimeric coiled coils, residue *g*(n) from one peptide faces residue *e*(n+1) from the other peptide. For the other positions we chose alanine (A) and glutamine (Q), which favor the α-helical conformation.

As a result of these considerations, the two basic coiled-coil units are:

ACC1:
abcdefg abcdefg abcdefg abcdefg
IAQLKWK NAALEQE IQQLKAK IAQLEAE

ACC2
abcdefg abcdefg abcdefg abcdefg
IAQLEWE IAALKQK IQQLEAE NAQLKAK

According to a coiled-coil prediction tool[2] (http://www.ch.embnet.org/software/COILS_form.html), the probability to form ACC1-ACC1 and ACC2-ACC2 coiled-coils is equal to 1. The different position of N, E and K in ACC1 and in ACC2 should prevent the formation of ACC1-ACC2 heterodimers as depicted in the following scheme (green and orange lines indicate favorable and unfavorable interactions, respectively).

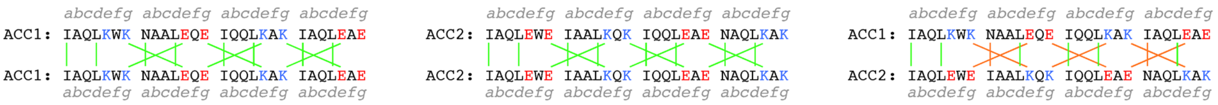

From these building units, the bioprobes are constructed from synthetic genes according to the scheme: *N*-membrane sensor region – linker – $(ACC)_{x4}$ – GFP (or mCherry)-*C*

The ACC1 or ACC2 unit is repeated four times to give a rod of about 12 nm (assuming a translation of 1.4Å per residue). The linker sequence is GSTAMEGEEGPD, where glycine, proline and negatively charged amino acids should stop the coiled-coil structure and prevents direct interaction with biological membranes in the absence of the upstream membrane-binding region. The first five residues of the linker correspond to BamH1 and ATG sites used for cloning.

The 51-91 sequence of Spo20 is:
MDNCSGSRRRDRLHVKLKSLRNKIHKQLHPNCRFDDATKTS, where the underlined residues correspond to the amphipathic region

The inverted Spo20 sequence is thus
MDNCSGSRRRDHLQKHIKNRLSKLKVHLRPNCRFDDATKTS, where the amphipathic region of Spo20 membrane sensor region is read from C to N.

In summary the two synthetic genes are:

Spo20-ACC1 :

GGATCCATGGACAATTGTTCAGGAAGCAGAAGACGTGATAGGCTACATGTGAAGCTTAAATCCTTGAGGAATAAAATCCA
CAAACAACTTCACCCAAACTGTCGGTTCGATGACGCCACTAAGACTAGTGGATCCGCCACCATGGAAGGTGAAGAAGGTC
CTGATATCGCTCAACTTAAATGGAAGAACGCAGCATTAGAACAAGAGATTCAACAACTTAAGGCTAAAATAGCCCAACTT
GAAGCCGAAATTGCACAACTTAAGTGGAAGAATGCAGCACTTGAGCAAGAGATACAACAACTTAAAGCAAAGATTGCTCA
ACTTGAGGCTGAAATTGCTCAACTCAAATGGAAGAATGCTGCTCTCGAACAAGAAATCCAACAACTCAAAGCCAAAATCG
CTCAATTGGAAGCTGAGATTGCTCAATTAAAGTGGAAGAACGCTGCTTTGGAACAAGAAATTCAACAATTGAAAGCTAAA
ATCGCACAACTTGAGGCAGAGGGCTCGCCGGTCGCCACCatg

BamHI
ATG

which codes for the following amino-acid sequence:

GSMDNCSGSRRRD**RLHVKLKSLRNKIHKQLH**PNCRFDDATKTSGSATMEGEEGPDIAQLKWKNAALEQEIQQLKAKIAQL
  ---------------Spo20(51-91)--------------/---linker--/-------heptad1----------

EAEIAQLKWKNAALEQEIQQLKAKIAQLEAEIAQLKWKNAALEQEIQQLKAKIAQLEAEIAQLKWKNAALEQEIQQLKAK
---/--------heptad2------------/---------heptad3-----------/---------heptad4----

IAQLEAEGSPVATM
----

where bold residues indicate the amphipathic region of the Spo20 membrane sensor.

invSpo20-ACC2 :

GGATCCATGGACAACTGCTCAGGGTCTCGAAGACGGGATCACCTGCAGAAGCACATCAAGAACCGTCTGAGCAAACTGAA
AGTGCATCTGAGGCCCAATTGTCGCTTCGATGACGCGACCAAGACATCCGGTAGTGCCACTATGGAAGGCGAAGAAGGAC
CTGACATTGCTCAACTCGAATGGGAGATAGCAGCCTTGAAGCAGAAAATCCAGCAACTCGAAGCTGAGAACGCTCAACTT
AAGGCCAAGATTGCACAGCTGGAGTGGGAGATAGCCGCACTCAAGCAGAAGATCCAGCAATTAGAGGCAGAAAACGCTCA
GCTTAAAGCGAAGATTGCCCAGTTAGAGTGGGAGATCGCGGCCCTAAAGCAGAAGATTCAGCAGCTGGAAGCAGAGAATG
CTCAGCTCAAAGCCAAAATCGCACAACTGGAATGGGAGATTGCTGCCTTGAAACAGAAGATCCAACAGCTTGAGGCTGAG
AATGCCCAGCTGAAAGCCAAAGGTTCGCCGGTCGCCACCatg

which codes for the following amino-acid sequence:

GSMDNCSGSRRRD**HLQKHIKNRLSKLKVHLR**PNCRFDDATKTSGSATMEGEEGPDIAQLEWEIAALKQKIQQLEAENAQL
---------------invSpo20--------------------/---linker--/-------heptad1----------

KAKIAQLEWEIAALKQKIQQLEAENAQLKAKIAQLEWEIAALKQKIQQLEAENAQLKAKIAQLEWEIAALKQKIQQLEAE
---/--------heptad2------------/---------heptad3-----------/---------heptad4----

NAQLKAKGSPVATM
----

where bold residues corresponds to the inverted sequence of the Spo20 amphipathic region.

1. Woolfson DN (2005) The design of coiled-coil structures and assemblies. Adv Protein Chem 70: 79-112.
2. Lupas A, Van Dyke M, Stock J (1991) Predicting coiled coils from protein sequences. Science 252: 1162-1164.
