# Supplementary figures and images for: Interaction of the Spo20 Membrane-Sensor Motif with Phosphatidic Acid and Other Anionic Lipids, and Influence of the Membrane Environment
Source: PLoS One. 2014 Nov 26;9(11):e113484. doi: 10.1371/journal.pone.0113484 (PMC4245137; doi:10.1371/journal.pone.0113484)

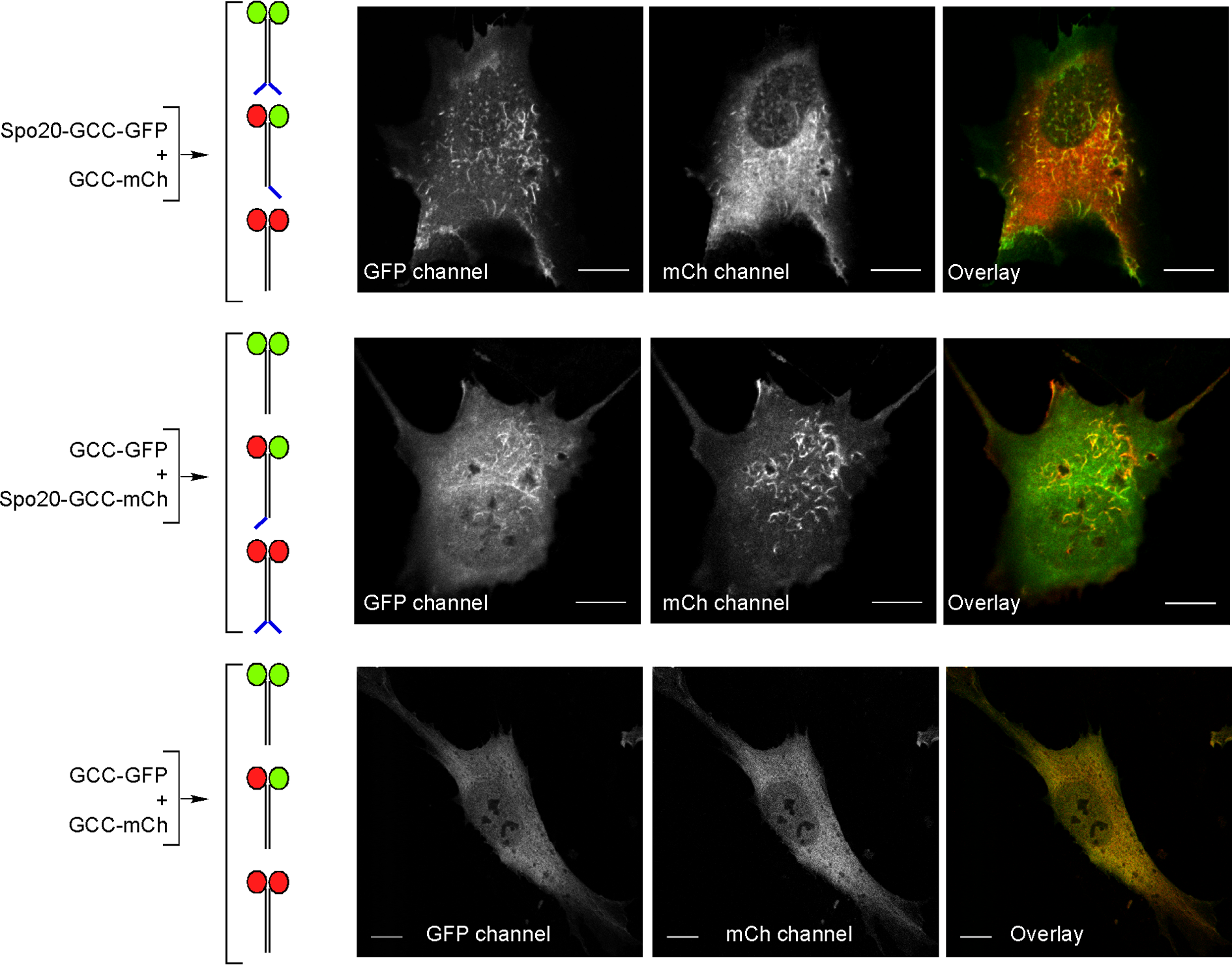

Supplement: Figure S1 — Subcellular localization of GCC bioprobes containing 0, 1 or 2 Spo20 membrane sensor regions. The experimental conditions were similar to that used in Fig. 3C except we used bioprobes containing GCC instead of ACC1 coiled-coil regions. The following pairs were coexpressed (from top to bottom): Spo20-GCC-GFP+GCC-mCherry, GCC-GFP+Spo20-GCC-mCherry, GCC-GFP+GCC-mCherry. The presence of the Spo20 membrane sensor region on one construct drives the membrane localization of the other construct. Scale bars, 10 µm. (TIF) [file pone.0113484.s002.tif]
